# Human and Animal Dirofilariasis in Southeast of France

**DOI:** 10.3390/microorganisms9071544

**Published:** 2021-07-20

**Authors:** Younes Laidoudi, Domenico Otranto, Natacha Stolowy, Sophie Amrane, Ranju Ravindran Santhakumari Manoj, Laurine Polette, Stéphanie Watier-Grillot, Oleg Mediannikov, Bernard Davoust, Coralie L’Ollivier

**Affiliations:** 1 IRD, AP-HM, MEPHI, IHU Méditerranée lnfection, Aix Marseille University, 19-21, Boulevard Jean Moulin, 13005 Marseille, France; younes.laidoudi@yahoo.com (Y.L.); drranjuvet@gmail.com (R.R.S.M.); olegusss1@gmail.com (O.M.); bernard.davoust@gmail.com (B.D.); 2 IHU Méditerranée Infection, Assistance Publique-Hôpitaux de Marseille, 19-21, Boulevard Jean Moulin, 13005 Marseille, France; sophie.amrane@ap-hm.fr (S.A.); laurine.polette@ap-hm.fr (L.P.); 3 Department of Veterinary Medicine, University of Bari, 70010 Valenzano, Italy; domenico.otranto@uniba.it; 4 Department of Pathobiology, Faculty of Veterinary Science, Bu-Ali Sina University, Felestin Sq, Hamedan 6517658978, Iran; 5 Hôpital de la Vision—La Timone, Assistance Publique-Hôpitaux de Marseille, 13005 Marseille, France; natacha.stolowy@ap-hm.fr; 6 French Military Health Service, Animal Epidemiology Expert Group, 37100 Tours, France; stephanie.watier@intradef.gouv.fr

**Keywords:** *Dirofilaria immitis*, *Dirofilaria repens*, electron microscopy, genotypes, France, dog, human, zoonosis

## Abstract

Dirofilariasis is one of the oldest known zoonotic infections of humans mainly caused by the filarial parasites of the species *Dirofilaria immitis* and *Dirofilaria repens*, which primarily infect dogs. A five-year survey (2017 to 2021) was conducted among the dog population to assess the molecular prevalence of *Dirofilaria* spp. in southeast France. Morphological and genetic analysis were performed on filaroids from dogs and one infected woman from the studied area. A total of 12 (13%) dogs scored molecularly positive for *Dirofilaria* spp. of which nine carried blood microfilariae. Ocular dirofilariasis was detected in a 79-year-old woman with no travel history. Both electron microscopy and molecular sequencing identified the worm in the human case as *D. repens*. Molecularly, *D. repens* isolates were identical in the human and dog cases, representing the only genotype reported so far in France. Despite the distribution of this genotype through all Europe, it was grouped separately with the other two European genotypes and with Asian ones. As in almost all previous human cases in France, *D. repens* parasites were mainly recovered from the ocular region of patients and were geographically concentrated in the southeastern regions. Data demonstrate the sympatric occurrence of *D. immitis* and *D. repens* with high risk of infection to human and dog populations in these investigated geographical areas, thereby underlining the urgent need to implement preventive chemoprophylactic strategies and vector control to reduce the risk of these filaroids in dog and human populations.

## 1. Introduction

The earliest recognized human infection with zoonotic filarial parasites was reported by Addario, who identified a filarial worm from the eyelid of an Italian woman as *Filaria conjunctivae* [1]. Later, Desportes (1939–1940) recognized that *F. conjunctivae* infection was actually caused by a species of *Dirofilaria*, which was named *Dirofilaria conjunctivae*. However, the history of human dirofilariasis dates back more than 400 years, when Amatus Lustianus described a clinical case of an eye worm infection in a three-year-old child in southern France, which is consistent with Addario’s description. That worm above has been redescribed as *Dirofilaria repens* in the Old World [2,3], causing subcutaneous and subcutaneous/ocular dirofilariasis in dogs and humans respectively [4]. Another zoonotic canine filaroid of the genus *Dirofilaria* is *Dirofilaria immitis* which occurs in many countries of the new and old world, being the etiological agent of cardiopulmonary dirofilariasis in dogs and humans [5,6]. *Dirofilaria immitis* was first reported from the hearts of hunting dogs in southern Europe, in Po River Valley of Italy, where Francesco Birago (1626) identified erroneously the parasite as *Dioctophyma renale*. Two centuries later (1856), the parasite was formally described by Joseph Mellick Leidy (1856) [7]. However, zoonotic potential of this parasite was not known until the infection with the same organism was discovered in humans in America during 1952 [7].

The genus *Dirofilaria* consists of two subgenera (i.e., *Dirofilaria* and *Nochtiella*) encompassing more than 27 valid and 15 questionable species that parasitize mainly carnivores and other mammals, including humans [8]. Due to the severity of disease and medical importance, *D. immitis* is of great veterinary importance while *D. repens* is the most commonly implicated species in human infections in the Old World [9]. These mosquito-borne filarioids share the same definitive hosts (canids) and insect vectors (i.e., Culicidae mosquitoes) [10]. These bloodsucking vectors feed indiscriminately on dogs and humans, leading to zoonotic sympatric occurrence in endemic areas [9], in particular, in Southern Europe, where both parasites are endemic. According to the report of Amatus Lusitanus, *Dirofilaria* infection seems to be present from the fifteenth century in France [2,3]. However, it is until the report of Panhot in 1952 regarding the infection of *D. immitis* in a native dog from Dombes (near Lyon), the presence of this parasite remains unnoticed in this geographical area. Since then, some reports on human and canine dirofilariasis have been documented. While information on the current active foci, the prevalence, and the threat posed to humans by these parasites in France remains scarce and dates mostly from four decades ago [11,12,13,14]. Some recent reports suggested the presence of an active focus of canine dirofilariasis in northern France [15,16], Corsica Island [17], and some autochthonous human cases caused by *D. repens* [18,19,20,21,22,23,24] and *D. immitis* [20,25]. In addition, a case of atypical subcutaneous and pulmonary dirofilariasis caused by *D. repens* has also been reported in a woman from this geographic area [26].

Recent advances in molecular taxonomy highlighted the presence of multiple genotypes and/or subspecies in both *D. immitis* and *D. repens* populations, suggesting that these filaroid species may be formed by a complex of species and/or subspecies. For example, the study of Yilmaz et al., (2016) based on the complete mitogenome analysis revealed the presence of considerable genetic variation between *Dirofilaria* (*Nochtiella*) species occurring in European and Asian countries, such as *Dirofilaria hongkongenisis* and *Dirofilaria* sp. “Thailand II” [27]. All these species are considered as the main causative agent of subcutaneous and/or subcutaneous/ocular dirofilariasis in dogs and humans respectively, from these areas. In addition, a particular genotype of *D. immitis* from South America differed molecularly from the classical genotype circulating worldwide. This genotype has been described as the more virulent strain of this species responsible for human ocular dirofilariasis in Brazil [6,28]. Despite these descriptions, limited information is currently available on the virulence, predilection sites within hosts, prevalence, and geographical assignment of these genotypes. Hence the present study was aimed to conduct a molecular surveillance of an active focus of canine dirofilariasis for a period of five years (from 2017 to 2021) and to perform molecular typing of canine and human filarioids of the genus *Dirofilaria* to better understand the epidemiological threat posed by these species in France.

## 2. Material and Methods

### 2.1. Sampling and Study Area

From 2017 to 2021, citrated blood samples were collected from a total of 92 dogs as per the recommendations of the Animal Ethics Procedures of French veterinarian forms and after getting the proper consent from the owners. All dogs (especially Belgian shepherds) were apparently healthy, received adequately veterinary care when needed, and were seasonally medicated with ectoparasiticides. The sampled dogs were between 2 and 10 years of age with an average of 5 years and were distributed within a 10-km radius around Miramas, France (43°35′27.2″ N 4°58′32.3″ E), a typical biotope focus (Figure S1) known to be endemic for canine dirofilariasis [11]. Of the total of 92 dogs sampled, 15 civilian dogs from the Haut Canadel (43°33′55.9″ N 5°01′47.9″ E), 33 military working dogs from the Miramas military camp (43°35′27.2″ N 4°58′32.3″ E), and 44 dogs from the Istres military airbase (43°30′45.2″ N 4°57′03.1″ E).

### 2.2. Case Report

**Figure 1:**
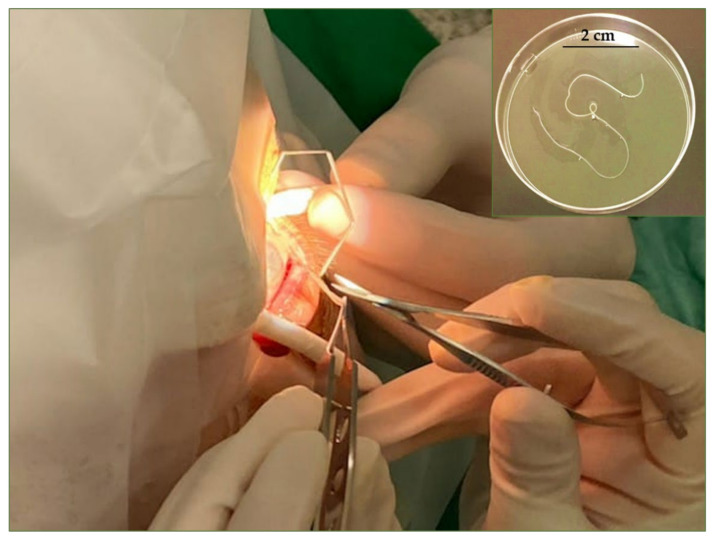
Close-up showing the extraction of worm coiled up in the subconjunctival tissue of the right eye.

A 79-year-old woman living in Miramas (43°35′27.2″ N 4°58′32.3″ E, Southeastern France) was admitted to the ophthalmology department at the La Timone University Hospital in Marseille, France, with a 10 day-history of pain in the right eye with an ocular discomfort and conjunctival injection. No ocular traumatism due to a fly bite or foreign body was mentioned by the patient. The slit-lamp examination showed a round translucent swelling mass under the bulbar conjunctiva of the right eye. Surgical excision of this mass was performed immediately, and a thin, whitish, cylindrical entire worm having a length of 10 cm was removed (Figure 1). Further examinations revealed no worm-like structures, and the eyelid and the retro- and peribulbar orbital area showed no significant pathological lesions. The patient had no travel history outside France. She owned a cat and a dog from the same region. When tested, these animals were amicrofilaremic and were *D. immitis* antigen test negative within the WITNESS^®^ Dirofilaria (Zoetis, Malakoff, France).

### 2.3. Morphological and Molecular Diagnosis of Dirofilaria spp.

All fresh canine blood samples were subjected to microscopic examination using the modified Knott’s test (Magnis et al., 2013) to identify microfilariae at the species level. Microscopic slides were examined under X40 magnification using a DM-LB2 microscope and Leica Las version 4.5.0 software (Leica 75 Microsystems, Wetzlar, Germany). The results of the laboratory diagnosis were immediately communicated to the owners or the concerned veterinarians in order to adopt appropriate treatments and control measures.

Macroscopic examination of the recovered whitish colored nematode from the human eye (Figure 1) was unsegmented and having a length of 10 cm and a diameter of 0.5 mm with tapered ends and a larger anterior end. A thin section of the removed worm was kept in 70% alcohol for further molecular analysis and the other part was then fixed in 4% buffered formaldehyde for more than 48 h and was sent to the PiCSL-FBI core facility (IBDM, AMU-Marseille) for transmission (TEM) and scanning (SEM) electron microcopic examinations.

Genomic DNA was extracted from all canine blood samples and from the recovered worm using the EZ1 DNA tissue kit (Qiagen, Courtaboeuf, France), following the manufacturer’s instructions. Two lysis steps were performed prior to the extraction procedures: (i) mechanical lysis performed on the FastPrep-24™ 5G homogenizer under high-speed agitation for 3 cycles of 40 s each in the presence of glass powder, (ii) enzymatic digestion of proteins with 200 µL of buffer G2 supplemented with 25% proteinase K for 24 h at 56 °C. The extracted DNA was eluted in a total volume of 100 µL and stored at −20 °C until molecular processing. Finally, all DNA from canine samples were screened for the presence of *Dirofilaria* spp. DNA using a species-specific multiplex real-time qPCR assay as described elsewhere [29].

### 2.4. Phylogenetic Analysis

Each single species-positive DNA sample (mono-infected dog) by the multiplex qPCR assay as well as the extracted DNA from the adult worm, were further subjected to the amplification with two standard pan-nematode PCR assays targeting 680 and 1127–1155 bps of the 12S and 18S genes respectively [15,30]. A third PCR [ITS-Nem] [30] was used to amplify 420–750 bps fragment of the Internal Transcribed Spacer 2 (ITS2) gene from the adult worm. PCR amplifications were performed in a thermal cycler (Applied Biosystem, Paris, France) as described elsewhere [30]. DNA amplicons were purified using NucleoFast 96 PCR plates (Macherey Nagel EURL, Hoerdt, France) prior to the sequencing reaction with the BigDye™ Terminator v3.1 Cycle Sequencing Kit (Applied Biosystems, Foster City, CA, USA). Big-Dye products were purified on Sephadex G-50 Superfine gel filtration resin and sequenced using 31 ABI Prism 3130XL sequencer.

DNA sequences were assembled and edited using ChromasPro software (ChromasPro 1.7, Technelysium Pty Ltd., Tewantin, QID, Australia) and then subjected to preliminary analysis using Basic Local Alignment Search Tool (BLAST) [31]. ITS2 sequences were aligned against all *Dirofilaria* ITS2 sequences available from the GenBank database. Sequence alignment was performed using the ClustalW application within BioEdit software [32]. DNA sequences from *Onchocerca borneensis* (MG192127) and *Onchocerca fasciata* (JQ316671) were used as outgroups to build the tree. The Tamura 3-parameter model (T92 + I) [33] was selected as the best-fitting nucleotide substitution model using the Akaike Information Criterion (AIC) option in MEGA6 software [34]. The maximum likelihood method (ML) based on 1000 bootstraps was inferred to generate the phylogenetic tree in MEGA6 [34]. Multi-loci sequence typing (MLST) phylogeny was performed based on the concatenated 12S and 18S DNA sequences. Briefly, 12S and 18S rRNA gene datasets were constructed from a representative member of canine, human, and zoonotic filaroids from previously published sequences [35], aligned with MAFFT [36] and concatenated with Seaview [37]. Sequences of *Filaria latala* (12S: KP760332; 18S: KP760135) and *Abbreviata caucasica* (12S: MN956811; 18S: MN956824) were used as out groups to root the tree. The ML phylogram was generated using IQTREE [38] with an ultra-fast bootstrap (UFBoot) of 10,000 replicates [39]. The best fitting evolutionary model (TIM3 *+ I + G4*) was selected using Modelfinder [40] (implemented as functionality of IQ-TREE). Analysis was performed on Galaxy Australia server [41]. All phylograms were edited using iTOL v4 software [42]. Interspecific pairwise distance (IPD) was used to estimate the evolutionary divergence between the ITS and the concatenated 12S and 18S DNA sequences among the Onchocercidae members. Standard errors were determined by a bootstrap procedure with 1000 replicates. Analyses were inferred using MEGA6 software, based on the Maximum Composite Likelihood model [34].

To elucidate the genetic variation among *Dirofilaria* (*Nochtiella*) species previously involved in human ocular/subcutaneous dirofilariasis and *D. repens* sequences obtained from the current study, the 12S sequences were aligned against an exhaustive sequence dataset (*n* = 75) retrieved from the GenBank database using BioEdit software [32]. This includes all available *Dirofilaria* (*Nochtiella*) species that cause canine and human dirofilariasis in the Old World. The sequence of *Dirofilaria* sp. (HQ540423) involved in human intraocular filariasis in the New World (Brazil) [43] was used as an outgroup to root the tree. Hasegawa-Kishino-Yano model was selected using the Akaike Information Criterion (AIC) option in MEGA6 software [34] to infer a 1000 bootstrap-based ML phylogeny on MEGA6 [34]. Finally, a Templeton–Crandall–Sing (TCS) network phylogram with a 95% connection limit and 1000 iterations was inferred using the PopArt software [44], along with the geographic assignment of each genotype.

## 3. Results

Of the total of 92 dogs screened by modified Knott’s test from different groups (i.e., civilian and military dogs from two different camps), at least one animal was found to be microfilaremic (i.e., 1 to 4700 microfilariae per ml of blood) with *D. immitis* (Figure S2a and supplementary file S1) and *D. repens* (Figure S2b and supplementary file S2) microfilaria in 7 (%) and 9 (%) dogs respectively. On molecular analysis, 7 (%), 3 (%), and 2 (%) dog blood samples scored positive for *D. immitis*, *D. repens* and both *Dirofilaria* spp. DNA, respectively. Among the *D. immitis*-positive dogs, two died during the period of study.

**Figure 2:**
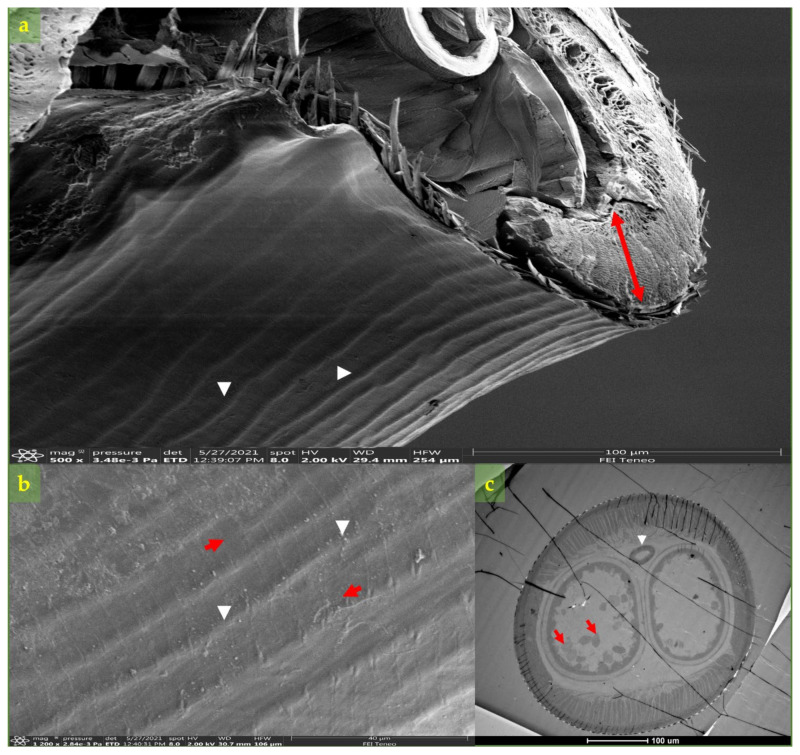
Worm morphology under SE and TE microscopy. (**a**) Cross-sectional tissue of body worm in SEM showing the longitudinal ridges (arrowhead) and, beneath the cuticle, a thick muscle layer (red arrow). (**b**) High-magnification image of the body surfaces under SEM showing the transverse striations (red arrows) and longitudinal ridges (arrowhead). (**c**) Ultra-thin cross-section by the TEM from the mid-body worm showing the intestine (arrowhead) and uterus filled with eggs (red arrows).

Scanning electron microscope (SEM) examination of the adult worm, from the woman’s eye, showed an outer thick, multilayered cuticle with longitudinal ridges and transverse striations (Figure 2a,b). Multilayered thickening beneath the cuticle a thick characteristic multilayered was observed (Figure 2a). Internally the intestine and a distended uterus filled with eggs were observed under the TEM microscope (Figure 2c). Based on morphology [45] and the ocular location of the parasite as well as molecular typing, the retrieved worm was identified as *D. repens*.

The 12S and the 18S PCRs yielded the amplification and sequencing of the target DNA from all examined samples (10 dogs mono-infected with *Dirofilaria* spp. and the worm DNA from the human case). All DNA sequences were deposited in the GenBank database under the following accession numbers: MZ435873-83, MZ427507-17, and MZ427935 for the 12S, 18S and ITS2 genes, respectively. Sequence analysis revealed that, all DNA sequences of *D. immitis*-positive dogs were 100 and 99.9–100% identical to the homologous 12S (AJ537512, KF707482, MT252024) and 18S (AB973231, MN795081, MN696499) respectively, from *D. immitis* circulating in the New and Old Worlds. While *D. repens* sequences from both dogs and the adult worm from the human case were identical to each other and showed and identity range of 96 to 100% for both the 12S (KX265092, KX265075) and the 18S (AB973231, MN696498) DNA sequences of *D. repens* circulating in Europe. In addition, the ITS sequence obtained from the adult worm showed 94 to 100% nucleotide identity with *D. repens* (KC429770, MH469230) from the Old World.

**Figure 3:**
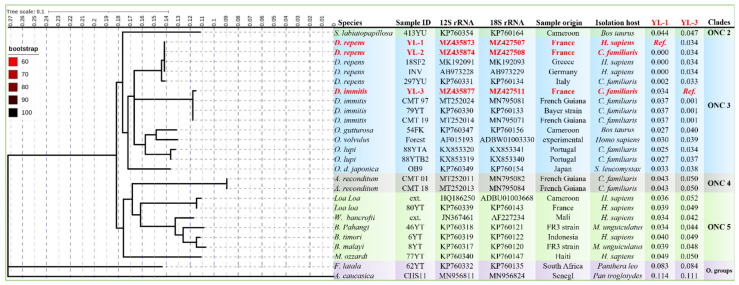
IQTREE generated from the concatenated (968 bps) sequences (*n* = 26) of 12S and 18S genes constructed from 10,000 bootstrap replication and TIM3 + I+G4 model. The tree shows the position of *Dirofilaria* spp. from the present study (indicated in red) among Onchocercidae Clades. The axis showed the global distance observed throughout the tree. Branches are color-coded according to the bootstrap’s percent. The identity of each taxa is color-coded according to the Onchocercidae Clades. Outgroup taxons are showing in grey. GenBank accession numbers, species name, host, and worm localization are indicated at each node. A total of 232 distinct site patterns were identified. Log-likelihood was −3851.749669. The number of base substitutions per site from between *D. repens* strain YL-1 and *D. immitis* strain YL-3 isolated in the present study and the other Onchocercidae members are shown in the last columns.

**Figure 4:**
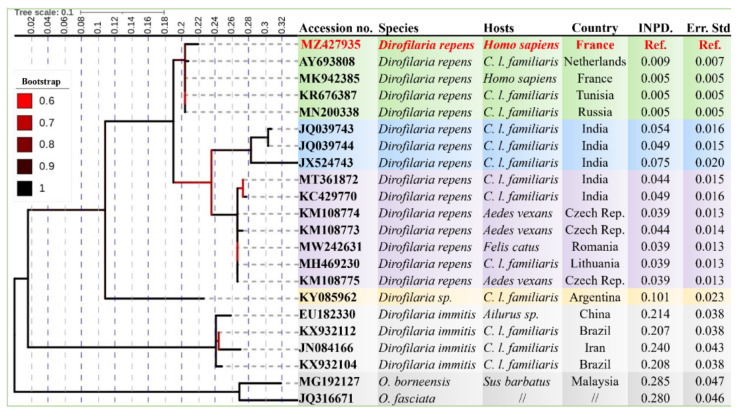
ML phylogram constructed by 1000 bootstrap replication and the Tamura 3-parameter model from the partial DNA sequences (340 bps) of the ITS. The tree shows the position of *Dirofilaria* spp. from the present study (indicated in red) among the other Dirofilaria strains. The axis showed the global distance observed throughout the tree. Branches are color-coded according to the bootstrap’s percent. The identity of each taxa is color-coded according to the genotypes of *Dirofilaria* spp. Outgroup taxons are showing in grey. GenBank accession numbers, species name, host, and geographical origin are indicated at each node. The numbers of base substitutions per site between *D. repens* worm isolated in the present study and the other isolates of *Dirofilaria* spp. are shown in the last columns.

**Figure 5:**
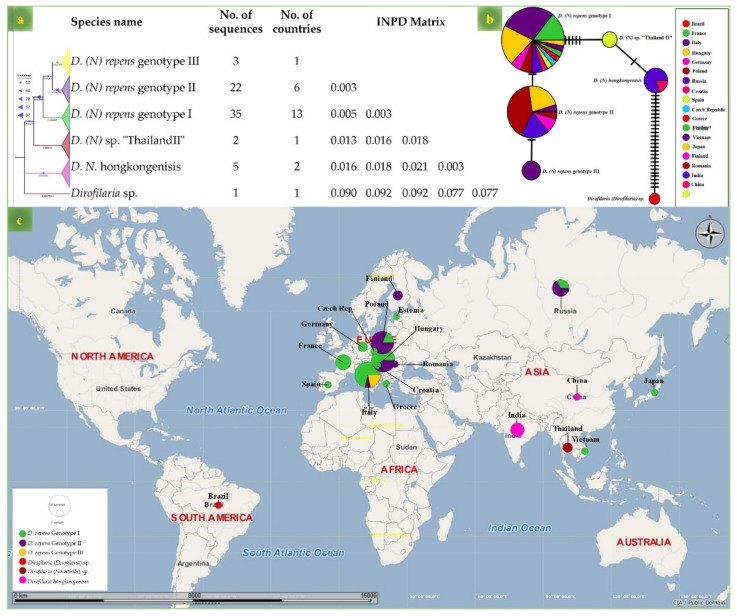
Genetic and geographical assignment of *Dirofilaria* (*Nochtiella*) species on the basis of 12S DNA sequences. (**a**) ML phylogram based on 75 DNA sequences of *Dirofilaria* (*Nochtiella*) species constructed from 1000 bootstrap replication and Hasegawa-Kishino-Yano model. The tree shows the position of the strain from the present study among *Dirofilaria* (*Nochtiella*) genotypes. (**b**) Templeton–Crandall–Sing (TCS) network phylogram elucidating the genetic diversity among *Dirofilaria* (*Nochtiella*) strains. (**c**) geographical assignments of *Dirofilaria* (*Nochtiella*) strains according to the 12S DNA sequences.

Both MLST and ITS phylograms showed that the genotype of *D. repens* herein isolated from the dogs and human patient, provisionally referred as “genotype I” is the one among the three genotypes circulating in Europe (Figure 3 and Figure 4), which is clearly different from *Dirofilaria* (*Nochtiella*) spp. circulating in Asia (Figure 4). Similarly, compared to the Asian strains, a lower INPD (0.005–0.009, Std. Err: 0.005–0.007) of the ITS gene was observed with the strains isolated from humans and dogs in Europe (MN200338, MK942385, AY693808) and North Africa (KR676387) (Figure 4) which was further strengthened by ML phylogeny and TCS network (Figure 5a,b) from the 12S DNA sequences of *Dirofilaria* spp. Furthermore, the ITS and MLST phylograms of the investigated *D. repens* from dogs and human formed a monophyletic clade with the genotype I circulating in all Europe excluding the other European (II & III) as well as the Asian genotypes (Figure 5c).

## 4. Discussion

This study suggests that *Dirofilaria* spp. infection may represent a threat to human and animal health in Southern France. It is worth noticing that the investigated dogs and the infected human had no travel history outside France. In addition, to confirm the previous report of human dirofilariasis in southern France [20], the present data provide information on the prevalence and molecular characterization of *Dirofilaria* spp. from dog and human populations in an old active foci, known to be endemic over the last four decades [11].

The overall prevalence of *Dirofilaria* spp. infection in canine population herein observed was 13.04% which was the same as the previous reported prevalence among military dogs from this area [11] and in northern France [15]. Presence of a typical biotope leading to the proliferation of Culicidae mosquitoes and the introduction of an invasive vector (*Aedes albopictus*) [46] along with the existence of potential wild reservoirs (e.g., foxes) in the vicinity of dog and human populations [47] may be a key factor for the perpetuation of these parasites under high prevalence in the studied area. Another possible reason could be the lack of year-round prophylactic measures for the proper vector control. The investigated dogs received only seasonal ectoparasite medication. However, due to the presence of year-round active vectors such as *Stegomyia albopicta* (a main vector of *Dirofilaria* spp.) in the southern regions of Europe [5,17], adoption of chemoprophylactic measures throughout the year is necessary to eliminate these vectors and thereby parasite transmission [48]. *Dirofilaria* transmission is related to an episystem complex involving several factors such as temperature, vector, host abundance, and the pathogen itself [9], which complicates disease control [49]. The European Society for Dirofilariosis and Angiostrongylosis (ESDA), the European Scientific Counsel Companion Animal Parasites (ESCCAP) and the American Heartworm Society (AHS) recommend strengthening preventive strategies through the combined use of anti-*Dirofilaria* larvae (macrocyclic lactone products) with vector prevention products (repellents) [50,51], as already demonstrated in field studies [52].

Despite adequate treatment, the death of two *D. immitis* positive dogs during the study period reflects the virulence of this parasite, an aspect which should not be ignored in the management of this disease. *Dirofilaria immitis* is responsible for cardiopulmonary dirofilariasis in dogs (known as heartworm disease) with varied symptoms such as exercise intolerance, fatigue to right heart, and lung failure [53]. On the contrary to this, *D. repens* infestation is often asymptomatic [53]. However, fatal outcomes of this diseases were observed in *D. repens* infestation, due to the massive release of the filarial endosymbiotic bacterium *Wolbachia* after microfilaricidal treatment [54]. Hence, in order to avoid fatal outcomes, these complications of antifilarial treatment have to be carefully considered while treating dirofilariasis [50,51,54].

**Figure 6:**
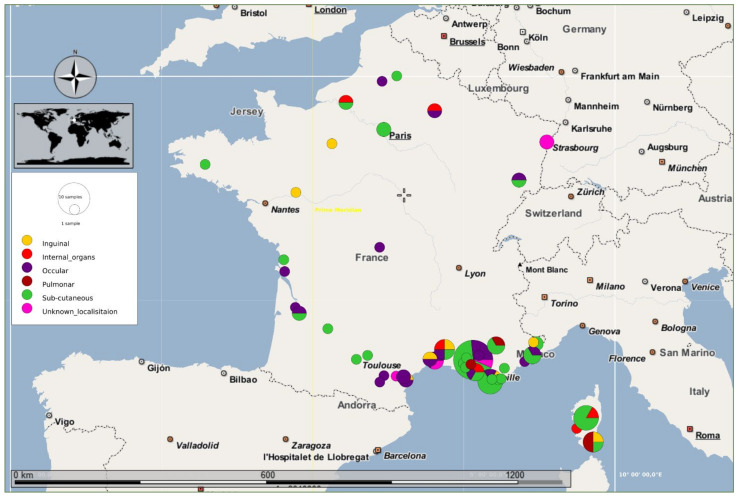
Geographical distribution of French human cases of *D. repens* infection reported so far. Cases are color coded according to the predilection sites of the parasite.

The only human case caused by *D. repens* recorded during the five years of surveillance in the studied area may not reflect the real incidence of human dirofilariasis, because of the lack of proper monitoring and the scant knowledge of physicians about the clinical presentation of the infestation in human patients. Since the first report of Amatus Lusitanus in 1566, 98 human cases of *D. repens* infection have been reported from France (Figure 6) [19,26,55], which place France in the second rank after Italy in the number of cases of human dirofilariasis [20]. Moreover, most of these cases (87%) were reported from the south of France. After carefully analyzing these facts, it appears that the human and canine populations have been at high risk for the past four decades in the studied area [11].

The management of vector-borne pathogens depends largely on the accuracy of diagnostic methods, so that optimal strategies for prevention, treatment, and control can be implemented [56]. However, in the case of *Dirofilaria* spp. infections, diagnosis is complicated and depends on several factors such as host species, predilection site, infection status, sex, and load of parasites [4]. Humans are dead-end host for *Dirofilaria* spp. and the parasites cannot reach the adult form, resulting in the microfilaremia appearing to be rare and incidental. These features delay detection of infection, and nodules are often confused with metastases on medical imaging. In addition, worm degradation takes place inside the nodules making morphological identification difficult [4]. In the present study, we used both electron microscopy and molecular based identification to identify the retrieved worm at the genotype level. However, despite the high specificity of the morphological and molecular identification tests we used here to diagnose canine dirofilariasis, these tests remain incomplete and are limited by the possible presence of occult infections among the studied dogs. The use of heartworm antigen detection and/or molecular detection of the specific genotype of the symbiotic *Wolbachia*, together with morphological and molecular identification of microfilaria, were required to avoid a false-negative diagnosis and thus to detect hidden reservoirs [29,57].

In contrast to *D. immitis*, mitogenome phylogenies of *D. repens* from humans and dogs revealed three phylogenetically distinct genotypes along with *D. hongkongenisis* and *Dirofilaria* sp. from Asian countries [27,57,58]. Genotype I and II of *D. repens* were sympatric in European countries, whereas genotype II was found only in Italy. The assessment of geographical prevalence based on genetic data could be a useful tool in monitoring the spread of this disease, as well as in identifying its origin when patient is without a travel history. For example, *D. repens* genotype I was also identified in Asia (Japan) in a woman with a travel history to Europe [59]. The strain involved in the human case reported in this study was the most dominant European genotype (I) and the only one reported to date in France. However, the occurrence of other genotypes in France cannot be excluded because of the lack of a broad DNA sequences database.

Current occurrence of human eye infection with *D. repens* reconfirms eye as a common predilection site of this parasite in humans [20]. According to the previous reports from France, *D. repens* were predominantly located in subcutaneous tissues (48%), with 50% of superficial nodules occurring in the head region. Ocular localization was second with a frequency of 26.5%. *D. repens* worms are thought to develop in the underlying tissues near the site of infection [20]. However, this hypothesis cannot be ruled out as the parasites were already found embedded deep in the body such as lungs (3.1%), genitals (8.7%), and internal organs (7.1%). The possible relationship between genotype of *D. repens* and the tropism of the parasite to different organs is still unknown due to the lack of strong molecular data. Seroepidemiological studies could be useful in assessing the exposure of human patients to both *Dirofilaria* spp. [60] considering that cases of human infestation usually overlap those in dogs [61].

## 5. Conclusions

Despite the epidemiological update on dirofilariasis in southern France, molecular surveillance of *Dirofilaria* spp. in both human and canine populations needs further research to accurately map the geographical prevalence of different genotypes. Conventional morphological identification coupled with molecular typing would be ideal for the prompt diagnosis of *Dirofilaria* spp. The current study has highlighted the intensity and infection pressure of *Dirofilaria* spp. infection for dogs and humans in southern France, drawing general attention to the public health risks. There is an urgent need to implement a well-designed preventive strategy against these zoonotic pathogens to limit their spread and the occurrence of human cases.

## Supplementary Materials

The following are available online at https://www.mdpi.com/article/10.3390/microorganisms9071544/s1, Figure S1: Photoshoot showing the typical biotope for mosquitos’ proliferation in Miramas, France. Figure S2: Modified Knott’s test showing (a) *D. immitis* and (b) *D. repens* microfilaria from canine blood. Supplementary file 1: alive *D. immitis* microfilaria (400×). Supplementary file 2: alive *D. repens* microfilaria (400×).
