# Supplementary figures and images for: Human and Animal Dirofilariasis in Southeast of France
Source: Microorganisms. 2021 Jul 20;9(7):1544. doi: 10.3390/microorganisms9071544 (PMC8307238; doi:10.3390/microorganisms9071544)

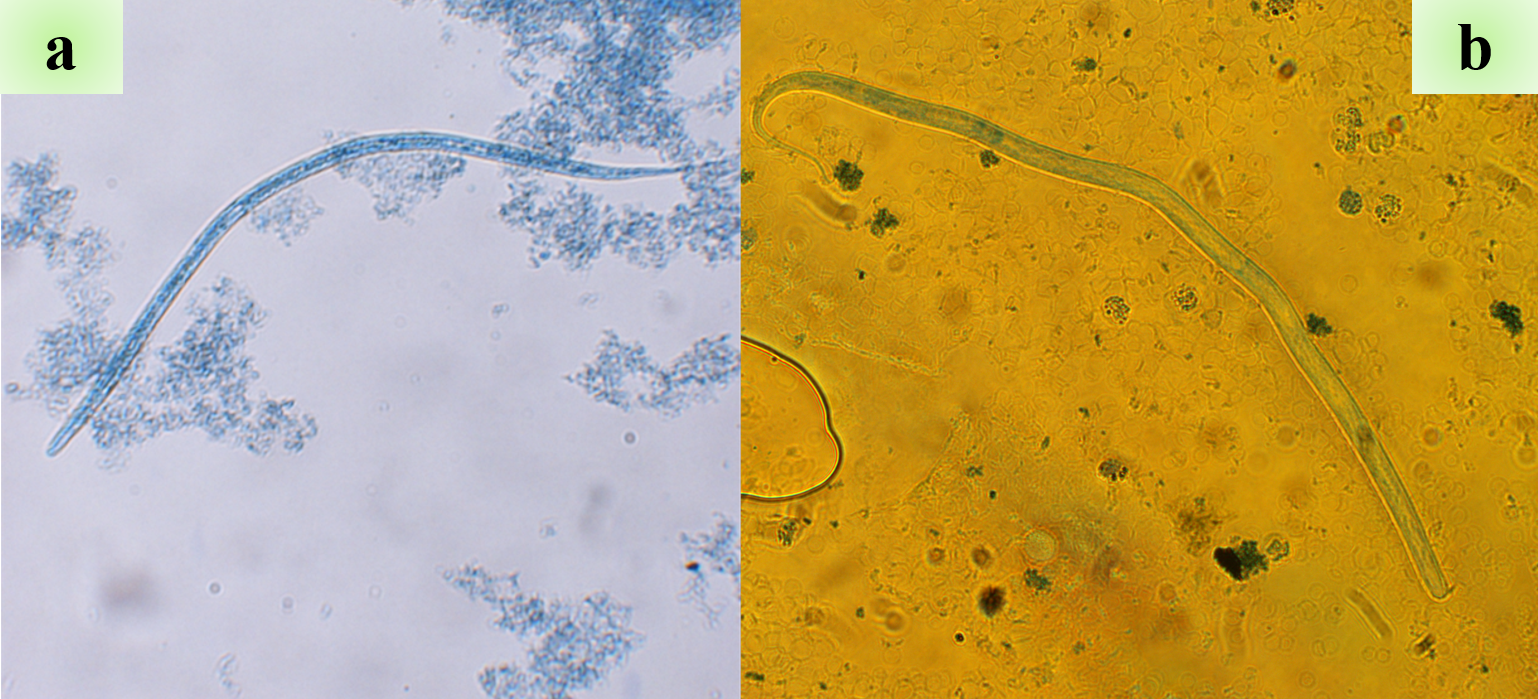

Supplement: Supplementary file 1 [file microorganisms-09-01544-s001.zip › Figure S2.tif]

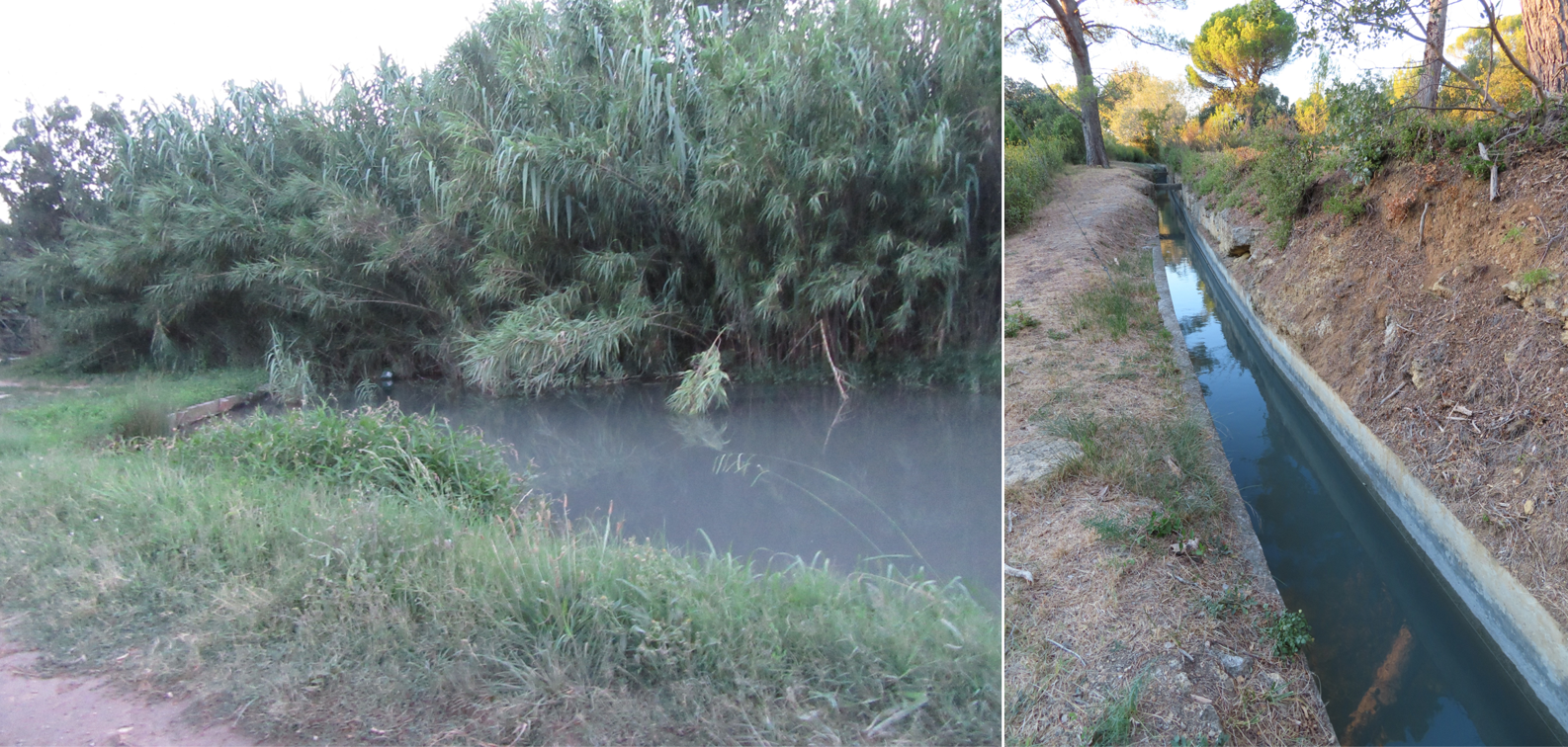

Supplement: Supplementary file 1 [file microorganisms-09-01544-s001.zip › Figure S1.tif]
